# Effect of Adhesion on Mechanical and Tribological Properties of Glass Fiber Composites, Based on Ultra-High Molecular Weight Polyethylene Powders with Various Initial Particle Sizes

**DOI:** 10.3390/ma13071602

**Published:** 2020-04-01

**Authors:** Sergey V. Panin, Lyudmila A. Kornienko, Qitao Huang, Dmitry G. Buslovich, Svetlana A. Bochkareva, Vladislav O. Alexenko, Iliya L. Panov, Filippo Berto

**Affiliations:** 1 Lab. of Mechanics of Polymer Composite Materials, Institute of Strength Physics and Materials Science SB RAS, 634055 Tomsk, Russia; rosmc@ispms.ru (L.A.K.); buslovichdg@gmail.com (D.G.B.); svetlanab7@yandex.ru (S.A.B.); vl.aleksenko@mail.ru (V.O.A.); 2 Department of Materials Science, Engineering School of Advanced Manufacturing Technologies, National Research Tomsk Polytechnic University, 634030 Tomsk, Russia; qz62368396@mail.ru; 3 Department of Mechanics and Graphics, Tomsk State University of Control Systems and Radioelectronics, 634050 Tomsk, Russia; panov.iliya@mail.ru; 4 Faculty of Engineering, Department of Mechanical and Industrial Engineering, Norwegian University of Science and Technology, 7491 Trondheim, Norway; filippo.berto@ntnu.no

**Keywords:** ultra-high molecular weight polyethylene, glass fiber, compatibilizer, adhesion, strength, wear resistance, permolecular structure, composition

## Abstract

The aim of this study was to assess the effect of adhesion between the non-polar, ultra-high molecular weight polyethylene (UHMWPE) matrix and the glass fiber fillers of various lengths treated with the commercially available “KH-550” agent, on the mechanical and tribological properties of the UHMWPE-based composites. The motivation was to find the optimal compositions of the polymer composite, for the compression sintering manufacturing of lining plates for the protection of marine venders and construction vehicles, as well as transport equipment. It was shown that the initial powder size at equal molecular weight determined the distribution patterns of the glass fibers in the matrix, and, as a consequence, the mechanical and tribological properties of the composites. Based on the obtained experimental data and the results of the calculation by a developed computer algorithm, control parameters were determined to give practical recommendations (polymer powder size and glass fiber length), for the production of the UHMWPE-composites having specified mechanical and tribological characteristics. The “GUR4022 + 10% LGF” composite, loaded with the chopped 3 mm glass fibers treated with the “KH-550”, was recommended for severe operating conditions (high loads, including impact and abrasive wear). For mild operating conditions (including cases when the silane coupling agent could not be used), the “GUR2122 + 10% MGF” and “GUR2122 + 10% LGF” composites, based on the fine UHMWPE powder, were recommended. However, the cost and technological efficiency of the filler (flowability, dispersibility) and polymer powder processing should be taken into account, in addition to the specified mechanical and tribological properties.

## 1. Introduction

Ultra-high molecular weight polyethylene (UHMWPE) possesses improved mechanical and functional properties, including a low friction coefficient, high resistance to wear, acids and alkalis, ultraviolet and gamma radiation. For this reason, UHMWPE is widely used in some industries and medicine [1,2,3,4,5,6,7,8,9,10,11,12,13]. Glass fibers are loaded in polymer composite materials, to increase their mechanical properties in numerous industrial applications [14,15,16]. However, the non-polar nature of UHMWPE results in low interfacial adhesion. Nevertheless, the treatment of glass fibers with silane coupling agents improves the adhesion between the components, in order to increase the mechanical and tribological properties of the UHMWPE-based composites [16,17,18].

The authors have studied the tribological and mechanical characteristics of the UHMWPE-based composites loaded with glass fibers (hereinafter referred “UHMWPE-based composites”), treated with conventional silane coupling agents [19]. It has been shown that the “KH-550” silane coupling agent (hereinafter referred to as “KH-550”) is the most efficient of them. The latter has significantly improved the tribological properties of the UHMWPE-based composites over a wide range of loads and sliding speeds. These studies have been performed using the UHMWPE-based composites fabricated from the “GUR-2122” fine powder (with a particle size of 5–15 μm). Meanwhile, a lot of papers have been published on the design of UHMWPE-based nano- and micro-composites from powders with larger particles [19,20] etc. However, the effect of the initial particle size of the polymer powder on the distribution of fillers and the mechanical properties of the UHMWP-based composites is not fully understood. Additionally, the influence of the fiber glass length on the mechanical and tribological characteristics of the UHMWPE-based composites has not been completely clarified. These data are relevant for their use as high-strength and wear-resistant lining plates, for the protection of marine venders, construction vehicles (excavator buckets and bulldozers), as well as transport equipment (dump truck bodies and railway cars for bulk cargo transportation, hydraulic locks, etc.).

The aim of the present study has been to assess the effect of adhesion between the non-polar UHMWPE matrix and the glass fiber fillers (of various lengths), treated with the commercially available “KH-550” agent on the mechanical and tribological properties of the UHMWPE-based composites. The glass fibers have been deliberately chosen as fillers for the following reasons:

(i) they have stable compositions, shapes and aspect ratios, so it has been possible to reliably compare the effect of micron and millimeter fiber lengths;

(ii) fiber glasses are a common low-cost industrial filler, characterized by a very high technological efficiency;

(iii) loading with the “KH-550” improves interfacial adhesion, which is one of the objectives of the study.

Methodological aspects of the research related to the fabrication and testing of samples are described in the Section 2 of the paper. The results of a comparative analysis of the mechanical and tribological characteristics of the UHMWPE-based composites fabricated from powders of different grades are presented in the Section 3. In the Section 4, the requirements for the UHMWPE-based composites have been justified on the basis of the reviewed papers and the manufacturer’s data, assuming their use as a structural material for lining plates. Control parameters in order to give recommendations (the sizes of the initial polymer powders and the glass fibers, silane-coupling treatment) for the fabrication of the UHMWPE-based composites, having the prescribed mechanical and tribological properties using the experimental data and a developed computer simulation algorithm, are defined in the Section 5.

## 2. Materials and Methods

Data on the powders (Celanese Corporation, Irving, TX, USA) and the glass fiber fillers used for the fabrication of the samples are presented in Table 1 and Table 2, respectively. Additionally, the “KH-550” silane coupling agent was used (3-aminopropyltriethoxysilane, chemical formula NH_2_CH_2_CH_2_CH_2_Si(OC_2_H_5_)_3_, produced by Dongguan First Rubber Plastic Technology Co., Ltd., Dongguan, Guangdong, China).

**Table 1:** The powders used for the fabrication of the samples.

Designation	Grade	Molecular Weight, Millions	Particle Size, μm	Crystallinity
Fine	Ticona GUR-2122	4.5	5–15 (weakly agglomerated into aggregates with a size of 120–150 μm)	Figure 1a
Middle size	“Ticona GUR-4120”	4.7	150	Figure 1b
Coarse	“Ticona GUR-4022-6”	4.7	330	Figure 1c

Powder size = ~5–15 µm, Crystallinity (χ) = 56.5%; Powder size = ~150 µm, Crystallinity (χ) = 30.5%; Powder size = ~330 µm, Crystallinity (χ) = 30.8%.

In order to remove the (technological) lubricant initially presented on the surface of the glass fibers, they were annealed in air in a “Memmert UF 55” oven (Binder, Tuttlingen, Germany) at a temperature of 300 °C.

The fiber glasses were treated with a water-ethanol (pH = 4.5–5.5) solution of the “KH-550” by continuous stirring using a “IKA C-MAG HS 7” magnetic stirrer (IKA Werke, Staufenim Breisgau, Germany), at room temperature for 30 min. The content of “KH-550” was 1% of the filler (0.1% of the total weight). Weighing was carried out suing a “SARTOGOSM LV 120-A” analytical balance (Sartogosm, St. Petersburg, Russia). Then, the suspension included the annealed glass fibers was dried in air using the “Memmert UF 55” oven at a temperature of T = 100 °C, until the liquid was completely evaporated.

The powders and the glass fiber fillers were mixed by dispersing the suspension in alcohol using a “PSB-Gals 1335-05” ultrasonic cleaner (“PSB-Gals” Ultrasonic equipment center, Moscow, Russia). The processing time was 3 min; the generator frequency was 22 kHz. After the mixing, the suspension was dried in the “Memmert UF 55” oven, using forced ventilation at a temperature of 120 °C for 3 h. The use of alcohol as a mixing medium suggested the absence of volatiles in the ready-made mixtures for hot pressing.

Bulk preforms of the composites were fabricated by the hot pressing of two-component powder mixtures at a pressure of 10 MPa and a temperature of 200 °C, using a laboratory setup based on a “MS-500” hydraulic press (NPK TekhMash LLC, Moscow, Russia). The setup was equipped with an open-loop ring furnace with digital temperature control (ITM LLC, Tomsk, Russia). After exposing under pressure, the preforms were cooled without unloading for 30 min. Cooling rate was 5 °C/min.

The tensile properties of the “dog-bone” shaped UHMWPE-based samples were measured under tension using an “Instron 5582” electromechanical testing machine (Instron, Norwood, MA, USA). The number of the samples of each type was at least four.

Wear resistance was evaluated according to the “block-on-ring” scheme, using a “2070 SMT-1” friction testing machine (Tochpribor Production Association, Ivanovo, Russia). Load on the samples was 60 and 140 N; (estimated) contact pressure P*_max_* was 9.7 and 32.4 MPa; sliding speed was 0.3 and 0.5 m/s. A counterpart was made of the outer ring of a commercial bear. It had a disk shape with a diameter of 35 mm and a width of 11 mm. The counterpart surface roughness was 0.20–0.25 µm. Wear rate was determined by measuring width and depth of the wear track according to stylus profilometry, followed by multiplication by its length. The wear rate values were calculated, taking into account the data on the applied load and the sliding distance:

Wear rate = volume loss (mm^3^)/sliding distance (m).

The wear track profiles were determined using the data on at least 10 tracks. Then, the wear rate values were estimated on the basis of the experimental test data over at least four samples of each type. Mathematical statistics methods were used for the experimental results processing.

Surface topography of the wear tracks was studied using a “Neophot-2” optical microscope (Carl Zeiss, Oberkochen, Germany), equipped with a “Canon EOS 550D” digital camera (Canon Inc., Tokyo, Japan), and an “Alpha-Step IQ” contact profiler (KLA-Tencor, Milpitas, CA, USA).

The cleaved surfaces of the notched specimens, mechanically fractured after exposure in liquid nitrogen, were used for permolecular structure studies. A “LEO EVO 50” scanning electron microscope (Carl Zeiss, Oberkochen, Germany) was employed (accelerating voltage was 20 kV).

Crystallinity was determined using a “SDT Q600” combined analyzer (TA Instruments, New Castle, DE, USA). IR spectra were obtained using a “NICOLET 5700” spectrometer (Thermo Fisher Scientific, Waltham, MA, USA) at the Shared Knowledge Center “Methods of Physical and Chemical Analysis” of National Research Tomsk Polytechnic University (Tomsk, Russia).

A “MI-2” abrasion testing machine (Metroteks LLC, Moscow, Russia) was used to determine volumetric wear values at abrasion by fixed particles, according to the “polymer pin-on-abrasive disk” scheme, regulated by the Russian State Standard GOST 426-77. The size of each of the two parallelepiped-shaped samples was 10 × 10 × 8 mm^3^. Wear rate was measured at a load of 6.8 N and a disk sliding speed of 17 m/min. Abrasives having a grit of P240, according to ISO 6344-3:2013, and a grain size of 50–63 μm were used. The samples were weighed using the “SARTOGOSM LV 120-A” analytical balance every 5 min of testing. Then, the volumetric abrasive wear rates were calculated by the formula:

$$\text{Wear rate}=\frac{\text{mass loss}\;\left(\mathrm{mg}\right)}{\text{sliding distance}\;\left(m\right)\times \mathrm{density}\;\left(g/{\mathrm{cm}}^{3}\right)}\times 1000$$

The topography of the wear track surfaces after abrasive tests was carried out using an optical interferential profilometer New View 6200 (“Zygo Corporation”, Middlefield, CT, USA).

## 3. Experimental Results and Discussion

The mechanical properties of the neat UHMWPE samples fabricated from the three powders listed in Table 1 and the UHMWPE-based composites containing 10% glass fibers (LGF and MGF, according to Table 2) after annealing, as well as after annealing and subsequent treatment with “KH-550” agent, are shown in Table 3, Table 4 and Table 5.

It is seen that the mechanical properties (elastic modulus and yield strength) of the composite, based on the fine GUR 2122 powder loaded with the annealed and treated glass fibers, almost doubled compared with neat UHMWPE, and was almost 1.5 times higher than the composite loaded annealed glass fibers (Table 3 and Figure 2a). Elongation at break (ε) decreased for the composites loaded with the annealed and treated glass fibers, but still remained high in both cases. Meanwhile, the elongation at break of the “GUR 2122 + 10% LGF” composite was the smallest, which corresponded to the maximum strength. Impact strength decreased for both composites (loaded with the annealed, as well as the annealed and treated glass fibers). The strongest composite had the lowest impact strength value of 54.4 kJ/m^2^.

**Figure 1:**
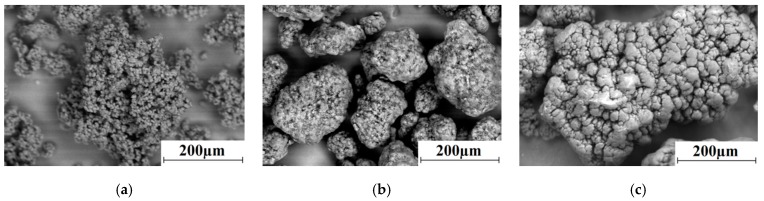
SEM micrographs of the Ultra High Molecular weight Polyethylene UHMWPE powders: GUR 2122 (**a**); GUR 4120 (**b**); GUR 4022-6 (**c**).

**Figure 2:**
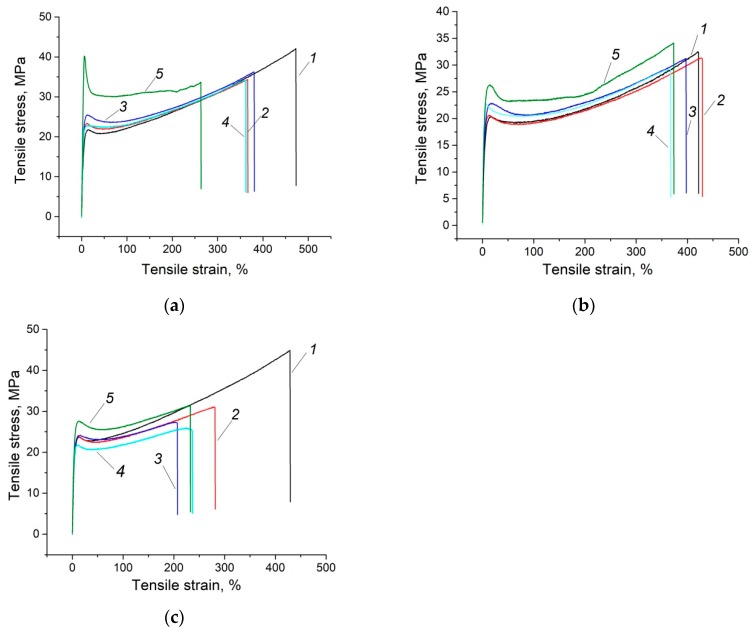
Engineering Stress-strain Curves for GUR 2122 (**a**); GUR 4120 (**b**); GUR 4022-6 (**c**): 1—neat GUR 2122; 2—“GUR 2122 + 10% MGF annealed” composite; 3—“GUR 2122 + 10% MGF annealed and treated with “KH-550”” composite; 4—“GUR 2122 + 10% LGF annealed” composite; 5—“GUR 2122 + 10% LGF annealed and treated with “KH-550”” composite.

Table 4 and Figure 2b indicate that the neat UHMWPE samples based on the “middle” size GUR 4120 powder had lower strength characteristics (elastic modulus, yield tensile strength, and ultimate tensile strength), compared with the composites based on the fine GUR 2122 powder with equal molecular weight. This was caused, among other things, by the lower crystallinity of the samples made from the GUR 4120 powder (χ = 30.5%, Figure 1). Accordingly, the composites based on this powder had lower strength properties, but increased impact strength. At the same time, the strength characteristics of the composites loaded with LGF and MGF after treatment with the “KH-550” increased by 1.2 times.

The mechanical properties of the neat UHMWPE samples, based on the coarse GUR 4022-6 powder, as well as the GUR 4022-6-based composites, are shown in Table 5 and Figure 2c. Firstly, the strength characteristics of the neat UHMWPE samples, based on the GUR 4022-6 powder were significantly higher than that for the previous two cases. Secondly, treatment of the filler with the “KH-550” improved the mechanical properties (elastic modulus and tensile strength) by ~1.3 times for the composite loaded with LGF. Moreover, its impact strength was rather high (92 kJ/m^2^). The results can easily be explained by the analysis of the formed permolecular structure.

**Figure 3:**
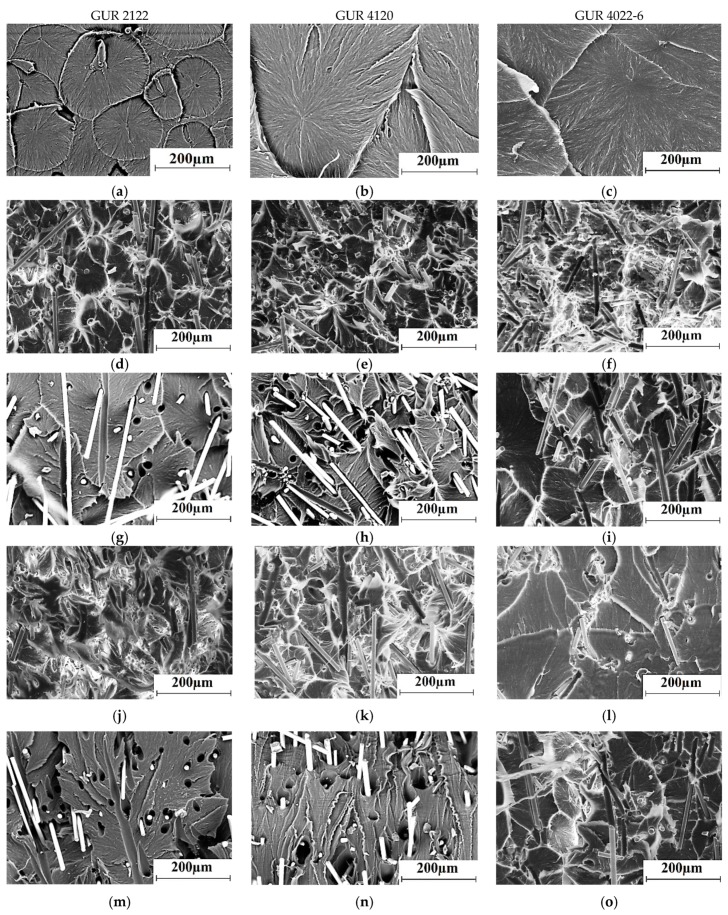
SEM micrographs of the sample permolecular structures: neat GUR 2122 (**a**), neat GUR 4120 (**b**), neat GUR 4022-6 (**c**); composites loaded with 10%: MGF annealed (**d**–**f**), LGF annealed (**g**–**i**), MGF annealed and treated with “KH-550” (**j**–**l**), and LGF annealed and treated with “KH-550” (**m**–**o**).

SEM micrographs of the permolecular structure of the neat UHMWPE samples, fabricated from the GUR 2122, GUR 4120, and GUR 4022-6 powders, as well as the glass fiber composites based on them, are shown in Figure 3.

The permolecular structure of the neat UHMWPE samples fabricated from all studied powders was spherulitic, with different sizes of structural elements (50–100 μm, 400–500 μm, and 600–1000 μm, respectively). Thus, the particle sizes of the initial UHMWPE powders had determined the sizes of the structural elements formed during the sintering and, as a result, the distribution of the fillers in the polymer matrix. The loading with the MGF resulted in the formation of a uniform finely dispersed spherulite structure (Figure 3d), but the loading with the LGF caused a less uniform filler distribution in the matrix (Figure 3g). The technological complexity of the LGF uniform dispersion was the main reason for this effect. Treatment of the glass fibers with the “KH-550” did not change the type of their distribution in the matrix very much (Figure 3g–i,m–o).

**Figure 4:**
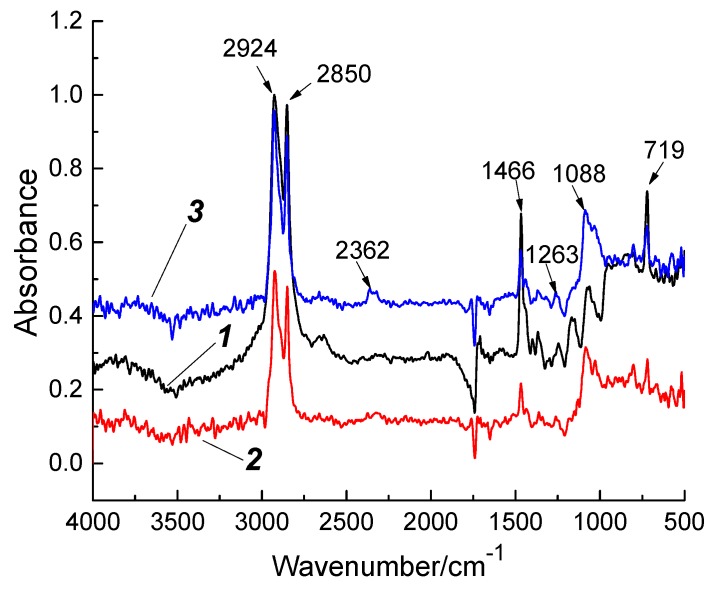
IR spectra: 1—neat GUR 2122; 2—“GUR 2122 + 10% LGF annealed” composite; 3—“GUR 2122 + 10% LGF annealed and treated with “KH-550”” composite.

The adhesion between the fillers and the polymer matrix as a result of the glass fiber treatment with “KH-550” was partially verified by IR spectra (Figure 4).

The strong absorption peak at 720 cm^−1^ and the relatively weak peak at 1243 cm^−1^ were the SiCH_2_ characteristic ones; at 800 and 1260 cm^−1^ were the Si(CH_3_)_2_ characteristic peaks, and at 1030 and 1080 cm^−1^ were the Si–O bond double absorption ones [16,21]. The IR spectra suggested that the intensities of the SiCH_2_ and Si(CH_3_)_2_ characteristic peaks increased for the composites contained the glass fibers treated with the “KH-550”, compared with the composites which contained the annealed glass fibers. The increasing of the SiCH_2_ and Si(CH_3_)_2_ peaks was determined by the chemical bond with the “KH-550”. The strongest characteristic peaks were at inverse wavelengths of 1263, 1088, and 2362 cm^−1^. This was governed by an increase in the number of Si–O bonds which occurred during the treatment of the glass fibers with the “KH-550”. Thus, the increase in the mechanical characteristics of the composites loaded with the treated glass fibers, in comparison with the composites loaded with the annealed glass fibers, was 1.3–1.5 times greater for all studied UHMWPE powders. This was due to the provided adhesion of the fillers with the matrix [16,21].

**Figure 5:**
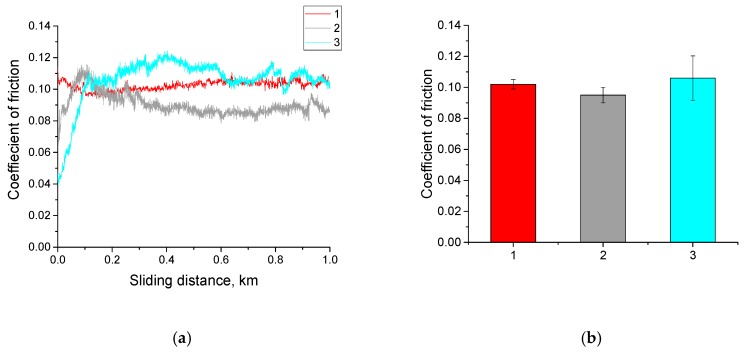
Diagrams on friction coefficients vs sliding distance during tribological tests (**a**) and its average values (**b**): 1—neat GUR 2122; 2—neat GUR 4120; 3—neat GUR 4022-6.

Furthermore, the tribological properties of all the above-mentioned composites were studied under the dry sliding friction and abrasive wear conditions. The friction coefficients for all types of the neat UHMWPE samples are shown in Figure 5. It is seen that all friction coefficient values depended weakly on the particle sizes of the initial powders, despite the fact that the samples had the different characteristic sizes of the structural elements with the same spherulitic permolecular structure.

**Figure 6:**
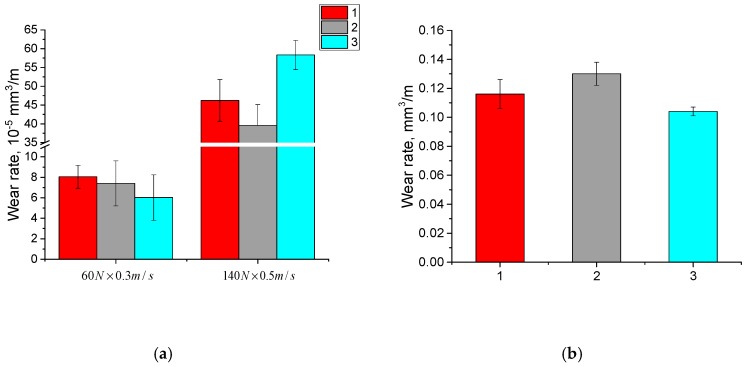
Diagrams on wear rates at dry sliding friction (**a**) and abrasive wear (**b**): 1—neat GUR 2122; 2—neat GUR 4120; 3—neat GUR 4022-6.

**Figure 7:**
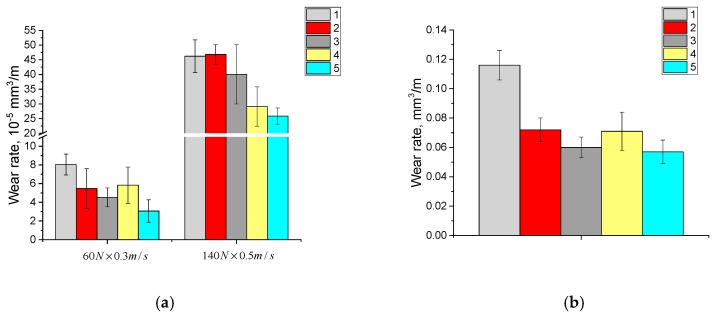
Diagrams on wear rates at dry sliding friction (**a**) and abrasive wear (**b**): 1—neat GUR 2122; 2—“GUR 2122 + 10% MGF annealed” composite; 3—“GUR 2122 + 10% MGF annealed and treated with “KH-550”” composite; 4—“GUR 2122 + 10% LGF annealed” composite; 5—“GUR 2122 + 10% LGF annealed and treated with “KH-550”” composite.

**Figure 8:**
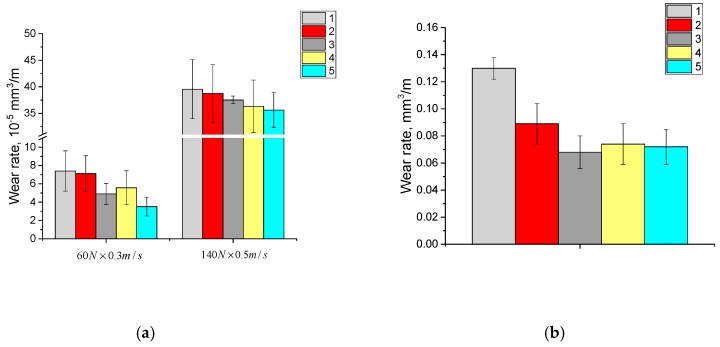
Diagrams on wear rates at dry sliding friction (**a**) and abrasive wear (**b**): 1—neat GUR 4120; 2—“GUR 4120 + 10% MGF annealed” composite; 3—“GUR 4120 + 10% MGF annealed and treated with “KH-550”” composite; 4—“GUR 4120 + 10% LGF annealed” composite; 5—“GUR 4120 + 10% LGF annealed and treated with “KH-550”” composite.

**Figure 9:**
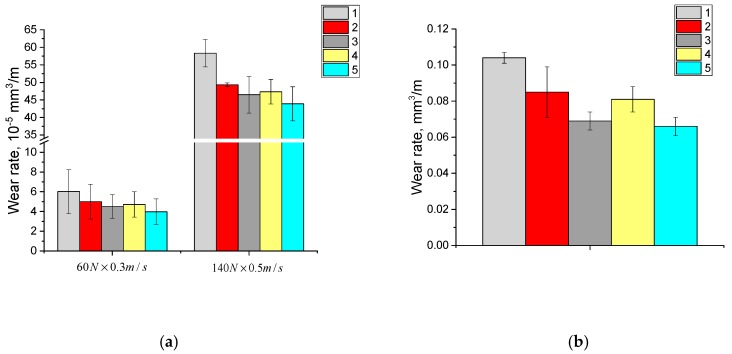
Diagrams on wear rates at dry sliding friction (**a**) and abrasive wear (**b**): 1—neat 4022-6; 2—“GUR 4022-6 + 10% MGF annealed” composite; 3—“GUR 4022-6 + 10% MGF annealed and treated with “KH-550”” composite; 4—“GUR 4022-6 + 10% LGF annealed” composite; 5—“GUR 4022-6 + 10% LGF annealed and treated with “KH-550”” composite.

The dependencies of the volumetric wear rate during dry sliding friction and the abrasive wear rate by fixed particles for the neat UHMWPE samples (Figure 6a,b) and the UHMWPE-based composites (Figure 7a,b, Figure 8a,b and Figure 9a,b) are illustrated in Figure 6, Figure 7, Figure 8 and Figure 9. It is seen in Figure 6a, that with increasing the particle sizes of the initial powders from 5 to 330 μm, the dry sliding friction wear rate of the neat UHMWPE samples decreased by ~10% under mild tribological loading conditions (60 N × 0.3 m/s) and increased under severe tribological loading conditions (140 N × 0.5 m/s). The abrasive wear rate increased by several times and only slightly depended on the initial powder particle sizes for all three neat UHMWPE grades (Figure 6b).

The volumetric wear rate for the composites, based on the fine GUR 2122 powder with different sizes of annealed glass fibers (LFG and MFG), was identical under the mild tribological loading conditions (60 N × 0.3 m/s) and decreases by 1.3–1.5 times for the composites loaded with the treated glass fibers. Likewise, the glass fibers treatment with the “KH-550” affected the wear resistance of the composites under severe tribological loading conditions (140 N × 0.5 m/s). The glass fiber length exerted practically no effect on the wear resistance of the composite fabricated from the fine GUR 2122 powder under abrasive wearing conditions. However, the glass fiber treatment with the “KH-550” to some extent increased its abrasion resistance (Figure 7b).

The dependence of the wear rate from the glass fiber length for the composites based on the “middle size” GUR 4120 powder was similar to the composites based on the fine GUR 2122 powder, both for the dry sliding friction and abrasive wear conditions (Figure 8a,b). At the same time, treatment with the “KH-550” increased the wear resistance of the composites loaded with MGF at abrasion by fixed particles, compared with the composites loaded with the LGF.

The wear rate of the composites based on the coarse GUR 4022-6 powder during dry sliding friction under the mild tribological loading conditions (60 N × 0.3 m/s) was similar to the ones based on the fine GUR 2122 powder, both in the wear resistance magnitude and the effect of glass fiber treatment with the “KH-550”. The glass fiber treatment with the “KH-550” also increased the wear resistance of the composites under severe tribological loading conditions (140 N × 0.5 m/s). This effect was highly pronounced during abrasive wear, when the wear resistance of the composites, based on coarse GUR 4022-6 powder, increased by 20%.

**Figure 10:**
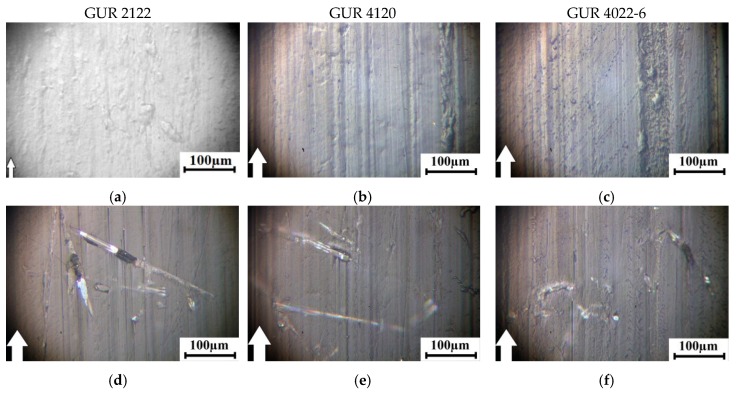

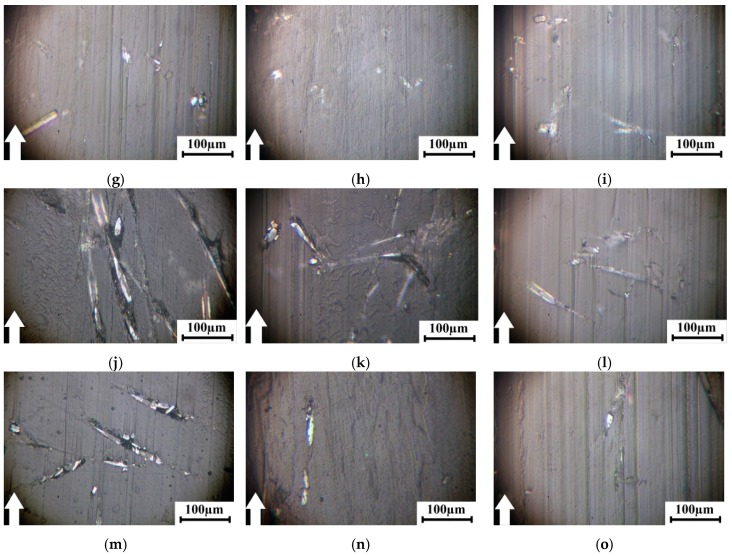
Optical images of the wear track surfaces of the neat Ultra High Molecular Weight PolyEthylene UHMWPE sample and its composites at 60 N × 0.3 m/s: based on the GUR 2122 powder (**a**,**d**,**g**,**j**,**m**); based on the GUR 4120 powder (**b**,**e**,**h**,**k**,**n**); based on the GUR 4022-6 powder (**c**,**f**,**i**,**l**,**o**).

**Figure 11:**
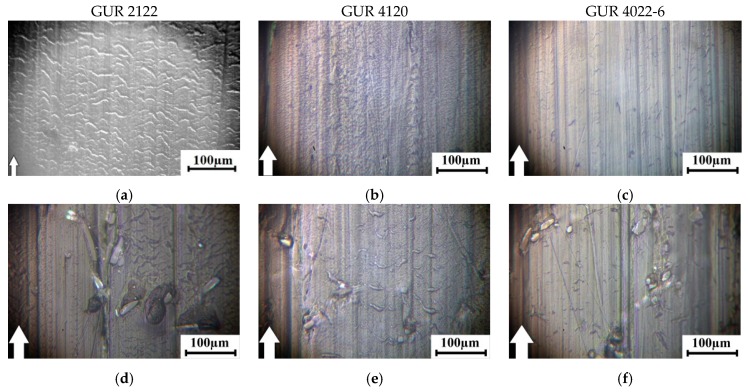

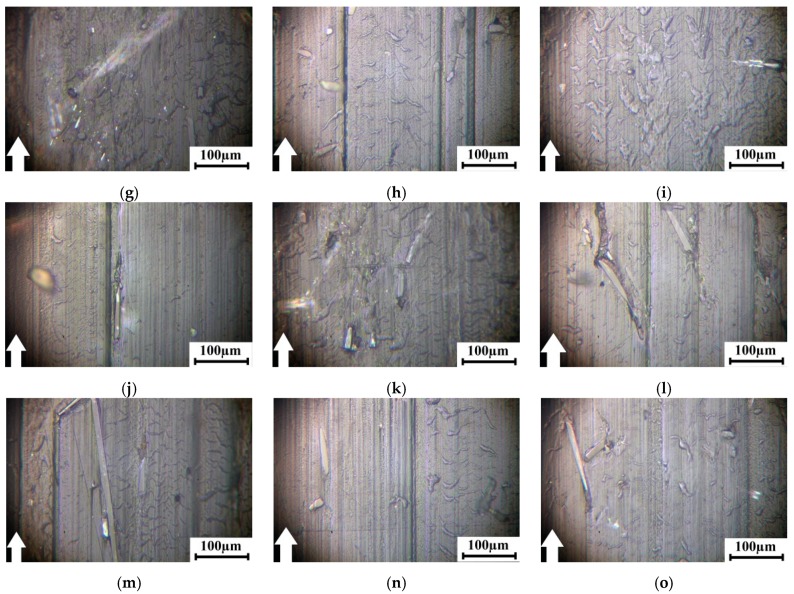
Optical images of the wear track surfaces of the neat UHMWPEE and its composites at 140 N × 0.5 m/s: based on the GUR 2122 powder (**a**,**d**,**g**,**j**,**m**); based on the GUR 4120 powder (**b**,**e**,**h**,**k**,**n**); based on the GUR 4022-6 powder (**c**,**f**,**i**,**l**,**o**).

The photographs of the wear surfaces of the samples after dry sliding friction of all the studied composites (Figure 10 and Figure 11) correlate well with data on their volumetric wear (Figure 6, Figure 7, Figure 8 and Figure 9). The sample surfaces of the composites loaded with the treated glass fibers were smoother after the dry sliding friction tests under mild (60 N × 0.3 m/s, Figure 9) and severe (140 N × 0.5 m/s, Figure 10) tribological loading conditions. On the one hand, this indicated that treatment with the “KH-550” enabled reinforcing fibers to be more firmly held in the polymer matrix. On the other hand, the wear resistance of the UHMWPE-based composites was provided, not only by the glass fibers protruded above the friction surface of the polymer matrix in the tribological contact, but also by improved mechanical strength, due to increased adhesion of the fillers with the polymer matrix.

**Figure 12:**
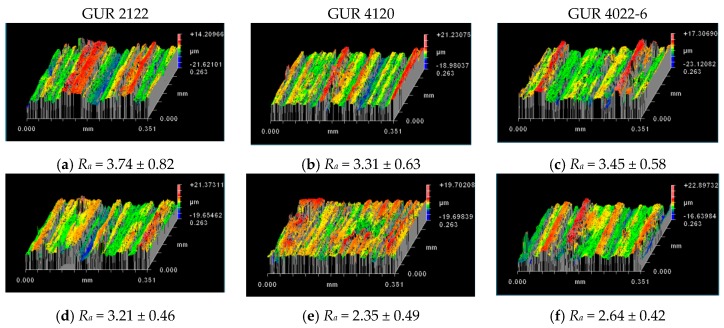

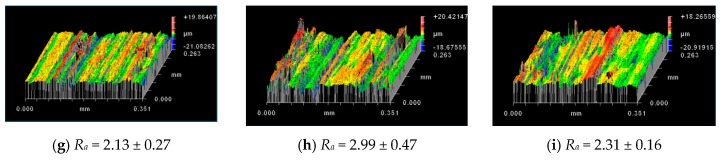
Optical profilometer 3D patterns of wear surface; abrasive wearing of neat UHMWPEE and its composites: neat GUR 2122 (**a**), neat GUR 4120 (**b**), neat GUR 4022-6 (**c**); composites loaded with 10%: MGF annealed and treated with “KH-550” (**d**–**f**), LGF annealed and treated with “KH-550” (**g**–**i**).

Filler adhesion with the matrix also determined the abrasive wear resistance of the UHMWPE-based composites (Figure 9b). The data in Figure 12 verifies that the composites loaded with the glass fiber treated with the “KH-550” had the highest abrasive wear resistance. Additionally, the surface of the composites loaded with the LGF treated with the “KH-550” possessed the smallest roughness Ra (Figure 12g–i).

The obtained mechanical and tribological characteristics of the UHMWPE-based composites, fabricated from the GUR 2122, GUR 4120, and GUR 4022-6 powders, loaded with glass fibers of various length (200 μm and 3 mm), enabled one to formulate recommendations on their use for the manufacturing lining plates for various purposes by the developed computer simulation algorithm (Section 5 below).

## 4. Justification of Requirements for the Properties of the Reinforced UHMWPE-Based Composites

A brief review of published papers on the research topic had been carried out before the justification of the numerical values of the specified physical-mechanical and tribological properties of the glass fiber UHMWPE-based composites for the manufacturing of lining plates. The review dealt with the following aspects:

(i) loading with fillers;

(ii) using of coupling agents;

(iii) optimal filler compositions to improve the tribological properties;

(iv) the effect of crystallinity and fabrication procedures on the polymer composite characteristics;

(v) the impact of lubricants on the wear mechanism;

(vi) the results of tribological tests at different temperatures, when ice was used as a counterpart.

These numerical data were then considered in Section 5, to specify the requirements for the properties of the glass fiber composites.

The abrasion resistance of the composites, based on high density polyethylene (HDPE) and Ultra High Molecular Weight PolyEthylene UHMWPE blends, was studied [22]. It was shown that the samples contained 30% UHMWPE possessed the minimum wear values. In this case, elongation to break was significantly reduced (down to 25%), but tensile strength increased (more than 20 MPa). Impact strength reached a constant level of about 70 kJ/m^2^ for the HDPE-UHMWPEE-based composites, with a UHMWPE content of 20%–30%. It should be noted that this characteristic was much higher for neat UHMWPE. However, it can be considered as a reference value.

In [23], adhesion was improved by the modification of wollastonite fibers with silane-titanate. It was shown that abrasive wearing was realized through plastic deformation, micro-plowing, cutting and cracking. The optimal weight fraction of wollastonite fibers provided almost twice as much abrasion resistance of the HDPE-UHMWPE-based composites, which tended to be 10%.

Additionally, investigations of the wear patterns of the UHMWPEE-based composites under severe conditions, specifically at high speeds and/or specific loads, were carried out [24]. It was shown that the UHMWPE wear rate was 3 × 10^−6^ mm^3^/N s, at high contact pressure during the initial stage of a tribological test (after 7000 revolutions according to the “ball-on-plate” scheme), while it decreased down to 5 × 10^−6^ mm^3^/N s after 85,000 revolutions, regardless of the surface roughness of the samples (0.06 or 0.53 μm). The friction tests were done using a SS440 steel ball used as a counterpart. Load was 30 N, the sliding speed was 4.5 m/min, the sliding distance was 4455 m. This fact was substantiated by decreasing the specific pressure on the polymer during tribological tests, that was estimated at 62.7 MPa, according to the Hertz theory. It was significantly higher than tensile strength. The authors suggested that the main mechanisms of the UHMWPE wear in such conditions had been plastic deformation and peeling. In this case, the sample had not been heated above 24 °C. However, this had been an integral temperature, without taking into account contact spots.

In [25], the effect of the UHMWPE crystallinity on the friction process, as well as wear resistance, was discussed. It was shown that, in the case of a higher crystallinity, the wear track depth decreased from 0.21 down to 0.12 μm, the groove depth after scratch tests decreased from 0.52 down to 0.46 μm (actually in the static loading–indentation mode), and the microscale friction coefficient decreased from 0.39 down to 0.28. Additionally, the interfacial shear strength increased from 7.13 up to 8.27 MPa. Thus, the use of finer UHMWPE powders, which enhanced the crystallinity of the UHMWPE-based composites, could also be an important “technological” factor for improving the tribological properties (wear resistance).

In addition, samples from the GUR 4120 powder loaded with basalt fibers, with a length of 2 mm and a diameter of 10 μm, were investigated [26]. The test scheme was “ball-on-disk”, the counter part was Si_3_N_4_ ball 6 mm in diameter; the test mode was dry sliding friction; the rotational speed was 2000 rpm. It was shown that the friction coefficient increased sharply from 0.06 up to 0.12 after 40 min of testing. It was about the same (0.06) at the rotational speed of 500–1500 rpm. The weight loss was 0.17 mg after 20 min of loading and 0.20 after 80 min, i.e., increasing linearly. The friction coefficient decreased from 0.06 down to 0.05, until the test time of 30 min, and then also gradually returned to its previous level, at loads of 50 and 100 N. At the high loads of 150 and 200 N, it continued to decrease slowly and reached about 0.04 after 80 min of tribological loading. The optimal content of basalt fibers was 10%. An increase in the tribological load contributed to the positive role of the fibers, due to the reinforcement effect. On the contrary, an increase in the rotation speed caused enhanced wear. Most likely, this was because of UHMWPE frictional heating and the low thermal conductivity of the basalt fibers.

In [27], UHMWPE with a molar mass of 6 million g/mol was studied. Basalt fibers with a length of 2 mm and a diameter of 12 μm were loaded. The fiber content ranged from 0% to 30 %. The surface roughness of the UHMWPE samples was 0.3 mm. The samples had the shape of a block with dimensions of 20 × 10 × 5 mm^3^. The counterpart, a ring of 40 mm diameter and 10 mm width, was made of the AISI-1045 steel. The counterpart hardness was 40–50 HRC and its roughness, Ra, was equal to 0.11–0.13 mm. The test condition was dry sliding friction. The load was 240 N, while the sliding speed was 0.42 m/s. The friction test time was equal to 120 min. It was shown that the impact strength slightly exceeded 90 kJ/m^2^ with a basalt fiber content of 10%. At the same loading degree, compressive strength was a little bit higher than 34 MPa. The friction coefficient was almost independent from the fibers loading (weight fraction), and was at the level of 0.27, even for neat UHMWPE at the established wear stage. The authors suggested that this value was due to a rather high load on the sample. Finally, the weight loss of neat UHMWPE was 15.5 mg, while it was reduced down to 1.5 mg with a basalt fiber content of 10%.

Additionally, UHMWPEE-based composites loaded with carbon fibers (CF) were investigated under both dry and distilled water lubrication conditions, using the “block-on-ring” scheme [28]. The block shaped samples and the 316 L stainless steel ring counterpart, with a diameter of 40 mm and a width of 10 mm, were used. The rotating speed was 200 rev./min, i.e., the sliding speed was 0.42 m/s. The load was 196 N and the test duration was 2 h. During the dry sliding friction test, the friction coefficient gradually increased from 0.17 up to 0.28 for neat UHMWPE, and it was at the level of 0.28 for the composites loaded with 20% CF (from the very beginning until the end). Volumetric wear gradually decreased from 1.9 mm^3^ for neat UHMWPE, to 0.7 mm^3^ for the UHMWPEE-based composites loaded with 30% CF. A similar trend was observed during lubricating friction. Volumetric wear nonlinearly increased from 0.6 to 2.7 mm^3^, with an enhancing load from 98 to 392 N (the growth was exponential under heavy loads). In terms of wear mechanisms, adhesion, ploughing, plastic deformation, and fatigue were the primary ones for neat UHMWPEE under dry sliding friction. In doing so, the worn surface exhibited a lot of micro-undulations. This effect was not observed for the UHMWPEE-based composited reinforced with CF. The dominant wear mechanism for these composites was evulsion of CF from the sample surfaces.

It was shown in [29] that the loading of the Derakane 441-400 (Epoxy Vinyl Ester, Dow Plastics) epoxy matrix (which was a vinyl ester-based resin), with reinforcement UHMWPE particles up to 30%, reduced the dimensionless abrasive wear rate, from 1.8 × 10^−8^ down to 0.7 × 10^−8^. An increase in the particle content was accompanied by enhanced deformation energy from 70 (without UHMWPE) up to 90 N·mm for the composites loaded with 30% UHMWPE. This indicated the ability of UHMWPE to absorb input energy. Given this assumption, the loading of UHMWPE with reinforcing fibers could facilitate the efficient transfer of load from the polymer matrix to the high-strength fibers, especially in the case of high adhesion. As a result, it was highly likely that resistance to the abrasive wear could be improved.

In [30], heat-resistant (with increased Vicat Softening Point) composites were designed, on the base of UHMWPE, loaded with recycled Polyamide 6 (R-PA6). The composites were fabricated through a melting extrusion route, using high-density polyethylene-graft-maleic anhydride (HDPE-g-MAH) as a compatibilizer. A twin-screw extruder (diameter was 30 mm, the length to depth ratio was 44) was employed at a constant speed of 160 rev./min and a temperature of 235–250 °C. It was shown that loading with 44% R-PA6 improved Charpi impact strength up to 33 kJ/m^2^. The paper [31] proposed a more advanced method for compounding HDPE and UHMWPE polymers by “solid-state shear pulverization SSSP”. Very high values of notched Izod impact strength (660–770 J/m) were obtained for the UHMWPE-based composites containing 30%–50% HDPE. The values were almost four times higher than those (170 J/m) for neat UHMWPE. In this case, however, yield strength decreased from 27 down to 21 MPa, in regard to neat HDPE.

It was shown in [32] that the basic mechanical properties of composites fabricated from two various UHMWPE powder grades (GUR 1020 and GUR 1050) by extrusion and compression sintering were different. For example, elongation was 452% for the GUR1020 extruded samples, while it was 440% for the molded ones. The difference was even more for the composites based on the GUR 1050 powders: 395% for the extruded samples and 373% for the molded ones.

The loading of UHMWPE with 10%–20% hard particles to improve bearing loading capacity resulted in decreasing the wear rate down to 35%–60% [33,34]. Additionally, the papers [35] recommended that the loading degree should not exceed 20%.

In [36], UHMWPE resistance to the ice abrasive wearing and the failure mechanisms were studied at different temperatures. It was shown that the friction coefficient decreased with the increasing test temperature. The load was 10 N and the sliding speed was 2.5 mm/s. The friction coefficient was at a level of 0.8–0.9 when the temperature was 258 K, while it tended to be zero at a temperature of 278 K. The reason was the ice melting and transformation to the lubricating medium. It was also found that the friction coefficient increased from 0.04 up to 0.16 (depending on the ice roughness), at a temperature of 268 K and a load of 10 N. Thus, the abrasive effect of ice could be physically simulated by fixed abrasive particle tests.

The following tribological test conditions were used in [37]: the test scheme was “pin-on-disk”, contact stresses were 3 MPa, the sliding speed was 200 mm/s, the sliding distance was 10 km, temperature was 37 °C. Pin-shaped UHMWPE samples were 25 mm long and 4 mm in diameter. UHMWPE had a molar mass of 3 million g/mol; its tensile strength was 35 MPa. A disk-shaped counterpart was made of alumina (HV 1800); it was 50 mm in diameter and 8 mm thick. The lubricationless friction coefficient was 0.13. However, it decreased down to 0.04–0.06 when saline, distilled water, or the blood plasma was used as the lubrication medium. The wear rate was 22 × 10^−8^ mm^3^/N·m for the dry friction conditions, 11 × 10^−8^ mm^3^/N·m for saline lubricant, 9 × 10^−8^ mm^3^/N·m for distilled water, and 5 × 10^−8^ mm^3^/N·m for the blood plasma. Additionally, it was shown that:

• a lot of fibrillar type debris was formed during the dry sliding friction (micro-adhesive wear) conditions;

• A lot of debris and grooves having particle shapes were found on the worn surfaces after the test in saline;

• many fatigue separated layers were evident on the worn surfaces after the test in distilled water;

• plow traces along sliding direction and a lot of deep microcracks normal to the sliding direction were observed on the sample surfaces, when the blood plasma had been used as the lubricant medium.

Thus, the environment and the lubricants had had a significant effect on the wear type, so the reinforcement aspects could have an important role in the long-term wear resistance.

The following is a brief overview of the quantitative indicators that various manufacturers cite as the main characteristics of lining plates.

A catalogue of the “Shibata Fender Team AG” company (Hamburg, Germany) [38] included fender panel facing, sliding boards, fender front shields, lock doors and entrances, quay wall protection, pile protection, etc. The properties of the material for their manufacturing are presented in Table 6. “The Rubber Company” (Hampshire, UK) [39] developed the “UHMW-PE-ECO” brand products, having the properties presented in Table 7. The “Polytech Plastics Australasia” (JANDAKOT WA, Australia), Ref. [40] also developed similar products (conveyor wear strips, guide rails, bearings, bushes, chute linings, pipe for the distribution of slurry materials, etc.) The properties of their products under the brand name “OMEGA-ULTRA^®^ Ultra-high molecular weight Polyethylene (UHMWPEE)” are summarized in Table 8. Many more examples could be given, but to complete the review, the characteristics of “Polystone^®^ M (UHMW-PE) Glass filled Röchling Engineering Plastics” glass filled composite plates [41] are given in Table 9. The plates were the structural material for truck bedliners, side rails and skirt boards, dragline bucket liners, etc. As another reference point, the authors consider to cite the manufacturer’s (one of the most famous companies of “Ticona LLC” [42]) data on one of the neat UHMWPE brands (Table 10).

Note that even in the area of the lining plate’s production, a significant range of the same operational properties is presented. The developers design and produce:

(i) oil-filled slabs for improved glide;

(ii) slabs to protect against sticking of ice and wet rock;

(iii) boards, mainly resistant to abrasion;

(vi) slabs to reduce slipping;

(v) slabs for marinas having high toughness and effective damping in the environment of sea water and salt fog, etc.

In this work, the authors did not set specific load-temperature operating conditions, but proceeded from the general purpose. The lining plates had to be with improved mechanical and tribological characteristics (due to the use of an industrially produced coupling agent), relative to neat UHMWPE. However, this approach could also be adapted to design other products with specified properties. Thus, based on the foregoing, the following quantitative indicators of the physical-mechanical and tribological properties were used as threshold values for Section 5:

• Impact strength was 75 kJ/m^2^. This value was half of the maximum value for the most impact-resistant of the studied neat UHMWPE samples (GUR 2122).

• Elastic modulus was 1000 MPa. This value exceeded the indicator for all investigated neat UHMWPE samples by 200–300 MPa.

• Yield strength for the treated and annealed fibers loaded composites was 21 and 24 MPa, respectively. In this case, the authors used different threshold values, since it was not possible to increase yield strength above 21 MPa for the used UHMWPE grades without ensuring proper interfacial adhesion.

• Tensile strength was 30 MPa. This value was slightly lower than that of the neat UHMWPE grades, since polymer reinforcement had been accompanied by a significant decrease in elongation to break, and, accordingly, in tensile strength. However, even for the neat UHMWPE grades, this value was quite high.

• The elongation at break was 215%. In published data, this parameter had been often set at 50% for the neat UHMWPE grades. In this case, the authors proceeded from the assumption that an increase in the strength properties due to glass fiber reinforcement should not be accompanied by a decrease in elongation to break by more than half in regard to the least deformable UHMWPE grade (the GUR4022-6 that had showed about 430%).

• The friction coefficient was 0.14. As published, the latter is weakly correlated with wear resistance. In this case, based on the assumption that loading with reinforcing fibers and improved wear resistance were rarely accompanied by a decrease in the friction coefficient, the threshold value was taken to be 40% higher than that for all studied neat UHMWPE grades.

• Volumetric wear for the treated and annealed glass fiber loaded composites (at P = 60 N and V = 0.3 m/s) was 5.9 × 10^−5^ and 5.0 × 10^−5^ mm^3^/m, respectively. Here, the authors again used different threshold values, due to the lower tribological properties of the composites loaded with the annealed fibers. They were chosen to provide higher wear resistance with respect to neat UHMWPE grades, which was about 20% for the treated fibers.

• Volumetric wear for the treated and annealed glass fiber loaded composites was 50.0 × 10^−5^ mm^3^/m at P = 140 N and V = 0.5 m/s. The used severe tribological loading conditions were accompanied by a much higher wear rate compared to the previous case and should be considered as briefly acting. Therefore, the authors chose a threshold value which was comparable with the wear resistance of most of the studied neat UHMWPE grades.

• Abrasive wear was 0.085 mm^3^/m. The threshold value was set to ensure improved resistance against fixed abrasive particles by at least 15%, relative to the most abrasion resistant GUR 4022-6 UHMWPE grade.

It should be noted that, conventionally, the threshold values of each of these parameters are assigned by a customer (based on specific requirements), as part of the technical specifications under real conditions for the designing of polymer composites. However, during the design, the designers are not limited to specific brands of polymers or fillers, but are free to choose them based on the task. It is important to achieve the specified physical-mechanical and tribological properties, while ensuring manufacturability and economic feasibility. In this work, the authors used a fixed set of components, when the size of the initial UHMWPE particles was varied with an approximately constant molecular weight of the polymer, while for the same filler content, the size of the fibers and adhesion were varied. Thus, the recommendations justified in this paper were based on the physical principles of the structure formation and the properties specified by them, rather than to obtain a specific composition for well-defined operating conditions. Nevertheless, the authors considered the results to be informative and shed light on the nature of the adhesion effect on the change in the physical and mechanical properties of the glass fiber-reinforced UHMWPE based composites.

## 5. An Algorithm for Designing a Composite for Lining Plates

Based on the results described in Section 3, a rational choice of the UHMWPE powder sizes and the length of the glass fibers was carried out, which provided the specified in ‘Section 4’ mechanical and tribological properties of the composites. For this purpose, a methodology had been developed to design the rational composition of the polymer composites [43,44,45,46]. The methodology had been verified in [43]. It was based on the use of a limited amount of experimental data (Table 3, Table 4 and Table 5 and Figure 2a–c, as well as the tribological test data in Figure 6, Figure 7, Figure 8 and Figure 9), which were supplemented by applying the interpolation procedure to obtain continuous dependencies. Then, the obtained dependences of the mechanical and tribological properties from the parameters of the components (in this case, the UHMWPE particle size and the length of the glass fibers) were drawn in the form of isolines. A region with the specified limits was identified, where all characteristic isolines overlapped each other. All the specified composite characteristics met in this region simultaneously. Calculated data visualization contributed to the assessment of the effect of the specified composite characteristics (control parameters) on the obtained properties. It made it possible for one to determine their specific values and to design the material with the specified properties.

In this case, the experimentally obtained characteristics (Table 3, Table 4 and Table 5 and Figure 7, Figure 8 and Figure 9) were determined by the particular UHMWPE powder sizes (Table 1) and the length of the glass fibers (Table 2; the length was zero without the fibers). They were the control parameters, while all other conditions were equal (loading degree of 10%, the fiber glass diameter, UHMWPE molecular weight, etc.) The additional values of the composite characteristics were calculated using interpolation [47], for intermediate points that determined the UHMWPE powder sizes (Table 1) and the length of the glass fibers (Table 2, the length was zero without the fibers). Since these parameters were interpolation nodes unequally spaced from each other, the Lagrange polynomial was used to determine intermediate values [43]:

$$P_{n}\left(x\right)=\sum_{i=0}^{n}y_{i}\left(\prod_{\begin{array}{c} k=0\\ k\neq 1 \end{array}}^{n}\frac{x-x_{k}}{x_{i}-x_{k}}\right)$$

where *x_i_*, *x_k_* were interpolation nodes; *y_i_* was function values in the interpolation nodes; *x* was an additional node. The more convenient form, as the Newton interpolation polynomial for unequally spaced nodes, could also be used [47].

The distance between the nodes that determined the length of the glass fibers (200 or 3000 μm; or zero without fibers) was incommensurable in the considered case. This fact resulted in a large interpolation error, even despite the use of the polynomial for the unequally spaced nodes [43]. In order to change the distance between the nodes and reduce the interpolation error, the values of the interpolation nodes were logged using the natural logarithm (ln 200 = 5.3 and ln 3000 = 8). The obtained values (5.3 and 8.0, respectively) were used as the nodal points.

**Figure 13:**
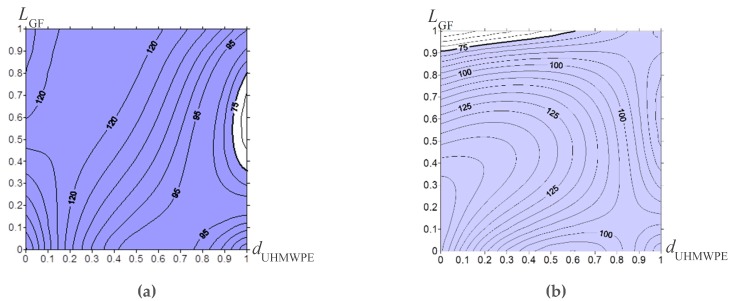

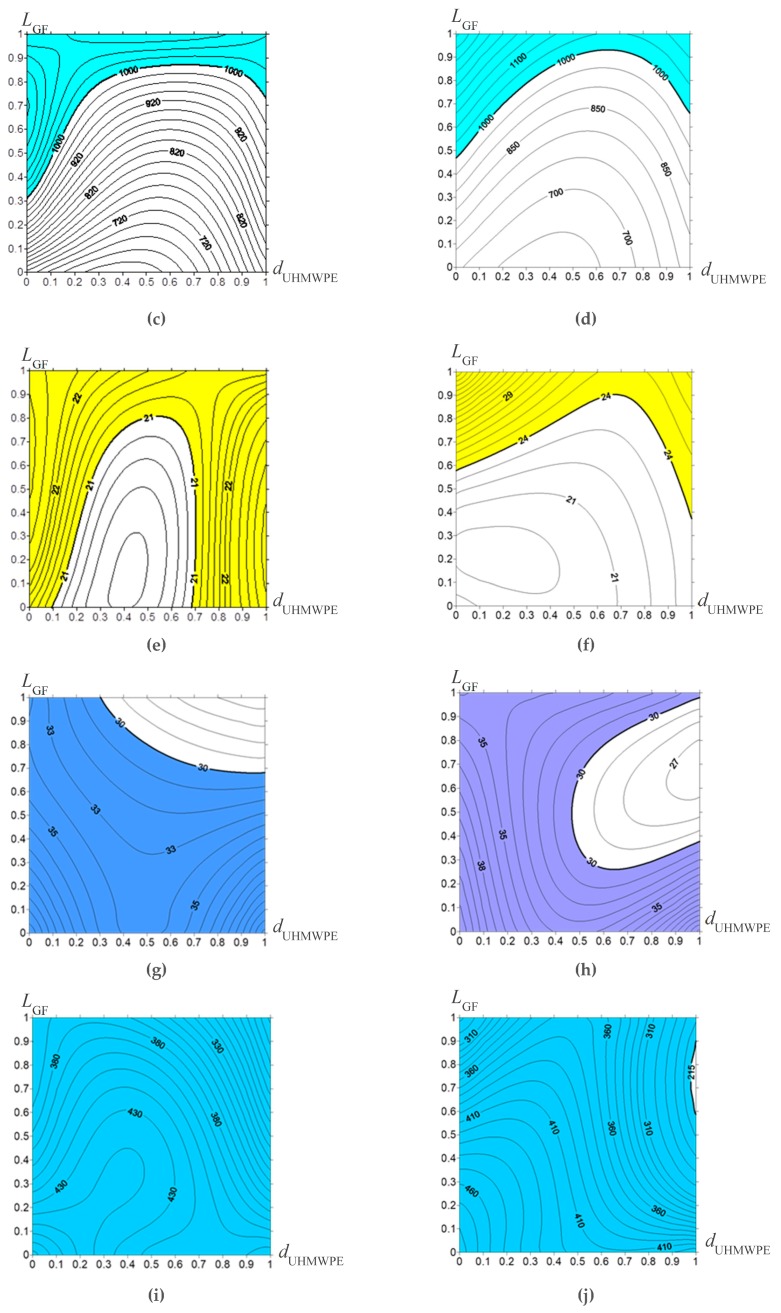

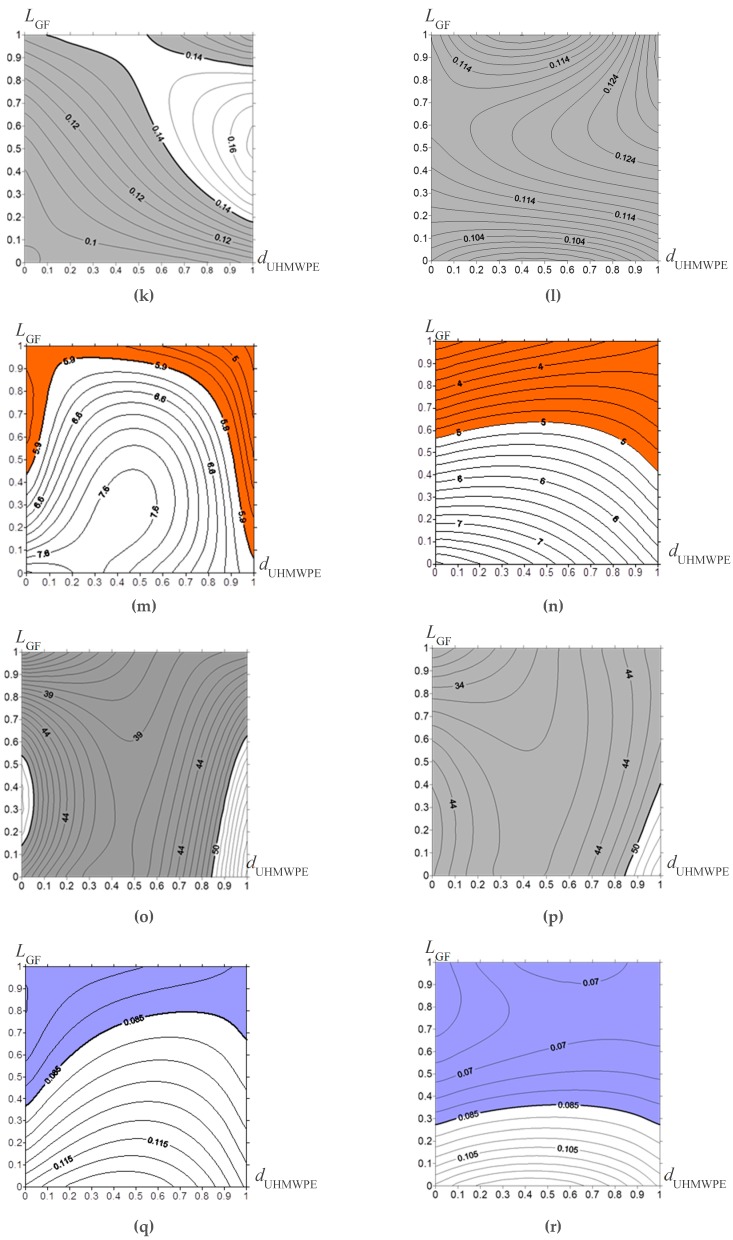
Isoline patterns on operational properties of the UHMWPE-based samples vs. particle sizes of the initial powders and length of the glass fibers without treatment (**a**,**c**,**e**,**g**,**i**,**k**,**m**,**o**,**q**) and after the treatment with “KH-550” (**b**,**d**,**f**,**h**,**j**,**l**,**n**,**p**,**r**): impact strength ak, kJ/m (**a**,**b**); tensile elastic modulus E, MPa (**c**,**d**); yield tensile strength σt, MPa (**e**,**f**); ultimate tensile strength σU, MPa (**g**,**h**); elongation at break ε, % (**i**,**j**); the friction coefficient f (**k**,**l**); the wear rate at 60 N × 0.3 m/s I, 10^−5^ mm^3^/h (**m**,**n**); the wear rate at 140 N × 0.5 m/s I, 10^−5^ mm^3^/h (**o**,**p**), the abrasive wear rate I, mm^3^/m (**r**,**q**).

When drawing the isolines for all composite characteristics (Figure 13), the values defining the UHMWPE particle sizes were plotted along the X axis, and the lengths of the glass fibers were along the Y axis. All nodal values were normalized from 0 to 1. It was necessary to combine the graphs if different nodal points were used for various characteristics. The region that met the specified limits for each operational property was highlighted in color.

**Figure 14:**
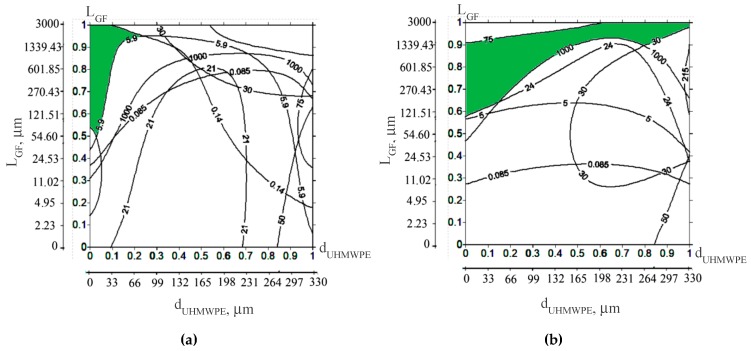
Areas that determine the rational diameter of the UHMWPE powder and the length of the glass fibers required to obtain compositions with the specified properties: without treatment (**a**), after the treatment with “KH-550” (**b**).

The intersected areas of all graphs and contours (Figure 13), after overlapping each other, are shown in Figure 13, for both composites fabricated without the treatment (Figure 14a), and after the treatment with the “KH-550” (Figure 14b). They determined the UHMWPE particle sizes and the length of the glass fibers required, to design the composites which have the specified properties. Figure 14 also shows the scale in micrometers corresponding to the (0–1) normalized scale, for the convenience of analyzing the obtained data.

It is seen, that in the case of untreated glass fibers (and a pair of slightly lower threshold values of their properties), only the composites based on the fine GUR 2122 powder loaded with the glass fibers of both lengths 200 and 3000 μm fell the “green” region (Figure 14a). This meant that any other glass fibers within this size range could be used as well. The main reason is determined by a uniform distribution of the glass fibers in the polymer matrix, which, even in the absence of chemical (van der Waals) adhesion, made it possible to obtain high mechanical and tribological properties. The use of the “middle” size, as well as coarse UHMWPE powders, did not enable one to have the specified composite parameters, mainly due to the less uniform distribution of the glass fibers and the insufficiently high mechanical properties.

When the glass fibers were treated with the “KH-550”, three composites (“GUR 4120 + 10% LCF + KH-550”, “GUR 4022-6 + 10% LCF + KH-550”, and “GUR 2122 + 10% MCF + KH-550”) fell the “green” region. It should be noted that the above selected “GUR 2122 + 10% LGF” composite (Figure 13a) did not reach the “green” region, even though its adhesion had been improved by the “KH-550” treatment. It is related to its limited impact strength: the increased strength properties (elastic modulus and yield strength) caused an almost three-fold decrease in impact bending resistance. On the contrary, the “GUR 4022-6 + 10% LCF + KH-550” composite, based on the coarse UHMWPE powder, did not have a significant decrease in impact toughness.

It is of interest that the “GUR 2122 + 10% MCF” composites loaded with the milled glass fibers fell the “green” region (Figure 14a,b) in both cases (without and after treatment with the “KH-550”). This result was caused by the formation of the homogeneous permolecular structure and the uniform filler distribution when the fine UHMWPE powder had been used. On the other hand, as noted above, the used UHMWPE powder size exerted a much lesser effect on the tribological than on mechanical properties. Therefore, the limited tribological characteristics of the composite (Figure 14a,b) did not affect their ability to reach the “green” region as much.

Based on the summarized above results, the “GUR4022 + 10% LGF” composite, loaded with the chopped 3 mm glass fibers treated with the “KH-550”, was recommended for severe operating conditions (high loads, including impact, abrasive wear). For mild operating conditions (including cases when the “KH-550” silane coupling agent could not be used), the “GUR2122 + 10% MGF” and “GUR2122 + 10% LGF” composites, based on the fine UHMWPE powder, were recommended. However, the cost and technological efficiency of the filler and polymer processing were also important factors, in addition to the mechanical and tribological properties.

In conclusion, the authors once again emphasize that the result described in the paper did not claim to design the particular composites for the well-defined operating conditions. The UHMWPE powders of three types having approximately the same molecular weight, the glass fibers of two sizes and one silane coupling agent were used. The most suitable for the production of the lining plates was chosen from them. In practice, the cost and technological efficiency (flowability, dispersibility) of the filler and polymer processing should be taken into account, in addition to the specified mechanical and tribological properties. The production cost could often play a decisive role in choosing a particular brand of polymer powders and fillers (which could be not only glass fibers). However, this does not change the essence of the proposed methodology to design the composites having the rational composition.

## 6. Conclusions

The analysis was made of the structure, the mechanical and tribological characteristics of ultra-high molecular weight polyethylene with different initial powder sizes (fine GUR 2122, “middle” size GUR 4120, and coarse GUR 4022-6), and the composites based on them, loaded with glass fibers of various lengths (200 μm and 3 mm).

It was shown that the permolecular structure of all three studied grades (GUR 2122, GUR 4120, and GUR 4022-6) was a spherulite one with different sizes of structural elements (50–100 μm, 400–550 μm, and 600–1000 μm, respectively). The dimension of the initial UHMWPE particles determined the size of the spherulites formed during sintering and, as a result, the distribution of the filler in the matrix. Loading with MGF (200 μm) resulted in the formation of the uniform finely dispersed spherulite structure. Loading with LGF (3 mm) caused its less even distribution in the matrix, primarily due to the technological complexity of its uniform dispersion. Treatment with the “KH-550” little changed the patterns of the LGF distribution in the matrix.

The strength characteristics (elastic modulus and yield strength) of the composites based on the fine GUR 2122 powder loaded with the treated LGF increased approximately twice, compared to neat UHMWPE, and one and a half times compared to the composites loaded with the annealed glass fibers. Elongation at break of both composites loaded with the annealed and treated glass fibers decreased, but was still high. However, impact strength of the composites decreased in both cases. It had the lowest value of 54.4 kJ/m^2^ for the highest strength “GUR 2122 + 10% LGF + KH-550” composite.

The neat UHMWPE samples, based on the “middle” size GUR 4120 powder, had lower strength characteristics (elastic modulus, yield tensile strength, and ultimate tensile strength) compared with the composites, based on the fine GUR 2122 powder with equal molecular weight. This was caused, among other things, by lower crystallinity of the samples made from the GUR 4120 powder (χ = 30.5%). Accordingly, the composites based on this powder had lower strength properties, but increased impact strength. At the same time, the strength characteristics of the composites loaded with LGF and MGF after treatment with the “KH-550” increased by 1.2 times.

The strength properties of the neat UHMWPE samples based on the coarse GUR 4022-6 powder are significantly higher compared to other studied GUR grades. The filler treatment with the “KH-550” improved the mechanical properties (elastic modulus and tensile strength) by ~1.3 times for the composite loaded with LGF. Moreover, its impact strength was rather high (92 kJ/m^2^). This result is related to uneven fiber distribution in a coarse spherulite polymer matrix.

It was shown that, with increasing the particle sizes of the initial powders from 5-15 to 330 μm, the dry sliding friction wear rate of the neat UHMWPE samples decreased by ~10% under mild tribological loading conditions (60 N × 0.3 m/s), and increased under severe tribological loading conditions (140 N × 0.5 m/s). The abrasive wear rate increased by several times and only slightly depended on the particle sizes of the initial powders for all three neat UHMWPE grades.

Based on the summarized results of all the studied physical, mechanical, and tribological properties, as well as the calculated data (by the methodology to design the rational compositions of the composites), the “GUR4022 + 10% LGF” composite, loaded with the chopped 3 mm glass fibers treated with the “KH-550”, could be recommended for the compression sintering manufacturing of lining plates for the protection of marine venders, construction vehicles (excavator buckets and bulldozers), as well as transport equipment (dump truck bodies and railway cars for bulk cargo transportation). However, the “GUR 2122 + 10% LGF” composites can be used, and silane coupling agent could not be used (for example, to prevent the adhesion of moist granular media at temperature extremes). Unfortunately, this would deteriorate the economic and technological efficiency of the composites.

Long glass fibers are more promising for reinforcing UHMWPE based composites, since the former can significantly improve the set of physical-mechanical characteristics and, to a much greater extent, provide increased wear resistance. LGF is more preferable for the manufacturing lining plates, due to their greater technological efficiency for the composite fabrication, lower cost, and good interfacial adhesion, after treatment with the “KH-550” silane coupling agent.

## Figures and Tables

**Table 2:** Fibrous fillers used for the composite fabrication.

Type	Mean Length, μm	Diameter, nm	Aspect Ratio	Manufacturer
MGF milled glass fiber (MGF)	200	9–14	20	GRAPHITE PRO Composition Technology Moscow, Russia
LGF long (chopped) glass fiber (LGF)	3000	9–14	300	GRAPHITE PRO Composition Technology Moscow, Russia

**Table 3:** Mechanical properties of GUR 2122 and GUR 2122-based composites.

Filler Composition, %	Density (*ρ*), g/cm^3^	Shore (*D*) Hardness	Elastic Modulus (*G*), MPa	Yield Strength (σ_Y_), MPa	Tensile Strength (σ_T_), MPa	Elongation at Break (ε), %	Impact Toughness (*a*), kJ/m^2^
None	0.928	57.5 ± 0.1	711 ± 40	21.6 ± 0.6	42.9 ± 3.1	485 ± 28	151 ± 6
10% MGF (200 µm) (annealed)	0.998	58.0 ± 0.3	1125 ± 39	23.2 ± 0.2	34.4 ± 1.4	366 ± 32	118 ± 9
10% MGF (200 µm) +KH550	0.994	59.2 ± 0.3	1154 ± 20	26.2 ± 1.5	37.3 ± 1.4	380 ± 43	119 ± 20
10% LGF (3 mm) (annealed)	1.011	60.3 ± 0.4	1038 ± 17	22.8 ± 1.2	34.2 ± 0.6	363 ± 22	113 ± 8
10% LGF (3 mm) +KH550	0.990	61.0 ± 0.4	1463 ± 95	40 ± 3.0	33.9 ± 3.7	263 ± 32	54.4 ± 5

**Table 4:** Mechanical properties of GUR 4120 and GUR 4120-based composites.

Filler Composition, %	Density (*ρ*), g/cm^3^	Shore (*D*) Hardness	Elastic Modulus (*G*), MPa	Yield Strength (σ_Y_), MPa	Tensile Strength (σ_T_), MPa	Elongation at Break (ε), %	Impact Toughness (*a*), kJ/m^2^
None	0.924	55.9 ± 0.6	624 ± 61	20.2 ± 0.8	33.7 ± 4.1	420 ± 33	93.2 ± 13
10% MGF (200 µm) (annealed)	0.991	55.9 ± 0.3	907 ± 96	20.7 ± 0.6	31.4 ± 0.3	424 ± 11	120 ± 3
10% MGF (200 µm) +KH550	0.990	57.7 ± 0.4	857 ± 59	22.5 ± 0.5	30.7 ± 1.6	397 ± 20	125 ± 14
10% LGF (3 mm) (annealed)	0.998	58.1 ± 0.6	1077 ± 132	21.7 ± 1	28.5 ± 1.9	367 ± 13	124 ± 20
10% LGF (3 mm) +KH550	0.993	59.2 ± 0.6	1094 ± 60	26.8 ± 1.5	33.9 ± 0.8	373 ± 16	68.5 ± 6.9

**Table 5:** Mechanical properties of GUR 4022-6 and GUR 4022-6-based composites.

Filler Composition, %	Density (*ρ*), g/cm^3^	Shore (*D*) Hardness	Elastic Modulus (*G*), MPa	Yield Strength (σ_Y_), MPa	Tensile Strength (σ_T_), MPa	Elongation at Break (ε), %	Impact Toughness (*a*), kJ/m^2^
None	0.938	57.9 ± 0.4	826 ± 45	23.7 ± 0.3	44.5 ± 3.5	429 ± 41	120 ± 4
10% MGF (200 µm) (annealed)	0.986	57.5 ± 0.3	989 ± 153	23.2 ± 0.7	30.3 ± 1	281 ± 18	68 ± 18
10% MGF (200 µm) +KH550	0.990	59.4 ± 0.1	1003 ± 93	25.1 ± 0.4	26.4 ± 1.8	207 ± 8.8	77 ± 17
10% LGF (3 mm) (annealed)	1.006	59.4 ± 0.6	1041 ± 29	21.3 ± 0.5	25.4 ± 2.6	237 ± 32	95.4 ± 9.5
10% LGF (3 mm) +KH550	0.992	59.8 ± 0.4	1149 ± 112	27.3 ± 1.7	30.4 ± 2.4	232 ± 15	92 ± 25

**Table 6:** Properties of the material used by the “Shibata Fender Team AG company”.

Properties	Values
Density, g/cm^3^	0.93–0.94
Friction Coefficient	0.15–0.20
Tensile Strength, N/mm^2^	≥17
Breaking Strength, N/mm^2^	>30
Break Elongation, %	≥50
Shore D Hardness	61–63
Ball Indentation Hardness, N/mm^2^	38
Impact Strength (with V-notch), mJ/mm^2^	≥70
Abrasion Sand Slurry Test	130

**Table 7:** Properties of the material used by “The Rubber Company”.

Properties	Values
Tensile Stress at Yield, MPa	20
Tensile Strain at Break, %	>50
Tensile Modulus of Elasticity, MPa	775
Charpy Impact Strength—Notched, kJ/m^2^	90
Shore D Hardness	60
Ball Indentation Hardness, N/mm^2^	34
Relative Volume Loss During a Wear Test in “Sand/Water-Slurry”	<160

**Table 8:** Properties of the material used by the “Polytech Plastics Australasia”.

Properties	Values
Impact Resistance (no brake), KJ/m²	179
Hardness D Shore	63
Friction Sliding Coefficient vs steel, MPa	0.18
Tensile Strength at Yield, MPa	20
Tensile Strength at Break, MPa	40
Elongation at Break, %	350
Modulus of Elasticity, MPa	650

**Table 9:** Properties of the material used by the “Polystone^®^ M” company.

Properties	Values
Density, mg/cm^3^	0.96
Tensile Strength, psi	2700
Elongation, %	265
Relative Volumetric Abrasion Lost *	75
Coefficient of Friction on Steel, static	0.15–0.20
Coefficient of Friction on Steel, dynamic	0.10–0.20
Izod Impact Strength, kJ/m^2^	110
Hardness D Shore	63–67

* Industry standard testing method using slurry of 60% aluminum oxide and 40% water, at a rotation speed of 1750 rpm for 2 h. Results indicate the ability of each material, in relation to Natural (=100), to resist abrasion under typical UHMW-PE applications. A lower number indicates better abrasion resistance.

**Table 10:** Properties of the material used by the “Ticona LLC” company.

Properties	Values
Density, g/cm^3^	0.926–0.934
Tensile Strength at Yield, MPa	21
Tensile Strength at Break, MPa	48
Elongation at Break, %	350
Young’s modulus, GPa	0.69
Izod Impact Strength (notch), kJ/m	1.6
Hardness D Shore	62–66
Abrasion Resistance	100
